# Comparison of the analgesic effect of ultrasound-guided paravertebral block and ultrasound-guided retrolaminar block in Uniportal video-assisted Thoracoscopic surgery: a prospective, randomized study

**DOI:** 10.1186/s12885-021-08938-7

**Published:** 2021-11-16

**Authors:** Qiang Wang, Shijing Wei, Shuai Li, Jie Yu, Guohua Zhang, Cheng Ni, Li Sun, Hui Zheng

**Affiliations:** 1 Department of Anesthesiology, National Cancer Center/National Clinical Research Center for Cancer/Cancer Hospital, Chinese Academy of Medical Sciences and Peking Union Medical College, No. 17, Panjiayuannanli, Chaoyang District, Beijing, 100021 China; 2 Department of Anesthesiology, National Cancer Center/National Clinical Research Center for Cancer/Cancer Hospital & Shenzhen Hospital, Chinese Academy of Medical Sciences and Peking Union Medical College, Shenzhen, 518116 China

**Keywords:** Lung cancer, Uniportal video-assisted thoracoscopic surgery, Pain, Ultrasound-guided paravertebral block, Ultrasound-guided retrolaminar block, Adverse events

## Abstract

**Background:**

The optimal modality for postoperative analgesia after uniportal video-assisted thoracoscopic surgery (UVATS) for the treatment of lung cancer has not yet been determined. Both ultrasound-guided paravertebral block (PVB) and retrolaminar block (RLB) have been reported to be successful in providing analgesia after UVATS. However, which block technique provides superior analgesia after UVATS is still unclear. This randomized study was designed to compare the postoperative analgesic effects and adverse events associated with ultrasound-guided PVB and RLB after UVATS.

**Methods:**

Sixty patients with lung cancer were randomized to undergo ultrasound-guided PVB (group P) or ultrasound-guided RLB (group R). In group P, 30 mL of 0.5% ropivacaine was injected at the T3 and T5 levels via ultrasound-guided PVB (15 mL at each level on the operative side). In group R, 30 mL of 0.5% ropivacaine was injected at the T3 and T5 levels via ultrasound-guided RLB (15 mL at each level on the operative side). The primary outcome was the numerical rating scale (NRS) score within 48 h after surgery. The secondary outcomes were total postoperative sufentanil consumption, time to first analgesic request and adverse events.

**Results:**

At 3, 6, 12, 24, 36 and 48 h postoperatively, the NRS score at rest in group P was lower than that in group R (*p* < 0.05). At 3, 6, 12, 24 and 36 h postoperatively, the NRS score while coughing in group P was lower than that in group R (*p <* 0.05). The total postoperative sufentanil consumption in group P was significantly lower than that in group R (*p* < 0.001). Additionally, the time to first analgesic request was longer in group R than in group P (*p* < 0.0001). The incidence of nausea in group R was higher than that in group P (*p* < 0.05).

**Conclusions:**

In patients with lung cancer undergoing UVATS, ultrasound-guided PVB with 0.5% ropivacaine provides better analgesia and results in less nausea than ultrasound-guided RLB. Compared with ultrasound-guided RLB, ultrasound-guided PVB seems to be a better technique for analgesia in UVATS.

**Trial registration:**

The name of this study is the Effect And Mechanism Of Ultrasound-guided Multimodal Regional Nerve Block On Acute And Chronic Pain After Thoracic Surgery. This study was registered in the Chinese Clinical Trial Registry (ChiCTR2100044060). The date of registration was March 9, 2021.

## Background

Lung cancer is by far the leading cause of cancer-related death in both men and women worldwide [1]. Surgical resection remains the main treatment for operable lung cancer patients [2]. However, lung cancer surgery can lead to severe postoperative pain [3]. With the popularization of video-assisted thoracoscopic minimally invasive surgery, the degree of postoperative pain has decreased compared with the degree of postoperative pain after open surgery, which is mainly due to the use of completely non-rib-spreading techniques [4]. Furthermore, with the widespread use of uniportal video-assisted thoracoscopic surgery (UVATS) techniques, postoperative pain is further relieved, which is mainly due to the minimal invasiveness of UVATS [5]. However, the postoperative pain of UVATS remains intense due to injury to the intercostal nerve, incisions in muscle and fascial tissue, and postoperative respiratory pain caused by thoracic drainage tube placement [5].

At present, the optimal modality for postoperative analgesia after UVATS has not yet been determined. Therefore, strategies to alleviate postoperative pain after UVATS with different analgesic techniques have been increasingly investigated [6]. Several ultrasound-guided thoracic nerve block techniques have been explored for perioperative analgesia in minimally invasive VATS, such as ultrasound-guided paravertebral block (PVB) and ultrasound-guided retrolaminar block (RLB) [6, 7]. PVB is a classic nerve block technique that has been shown to be effective in alleviating postoperative pain and opioid consumption after minimally invasive VATS [8]. However, there is a risk of unintentional iatrogenic pneumothorax and hydropneumothorax because the parietal pleura forms the anterior wall of the paravertebral space [9–11]. In addition, because the foramina form part of the medial wall of the paravertebral space, the risk of accidentally injuring the spinal nerve roots still exists [12]. Furthermore, with dual-plane or triple-plane block techniques, which are widely used in clinical practice, the risk of the aforementioned serious complications further increases [13]. Therefore, for novices, ultrasound-guided PVB is an advanced regional nerve block technique that requires a relatively long learning curve [12]. As a consequence, interest in finding effective, simpler and safer thoracic fascia block techniques continues. In recent years, ultrasound-guided RLB has been found to be effective in alleviating postoperative pain after thoracic surgery [14, 15]. Comparatively, due to the remoteness of the injection site from the pleura and spinal nerve roots and the superficial anatomical location, RLB is a relatively safe and easy nerve block technique for novice practitioners [14]. However, at present, it is not clear which of the two techniques has a better analgesic effect and is more suitable for postoperative analgesia in UVATS. Therefore, this prospective, randomized clinical study was performed to investigate which technique is better for postoperative analgesia in UVATS.

## Methods

### Trial design and inclusion and exclusion criteria

**Fig. 1:**
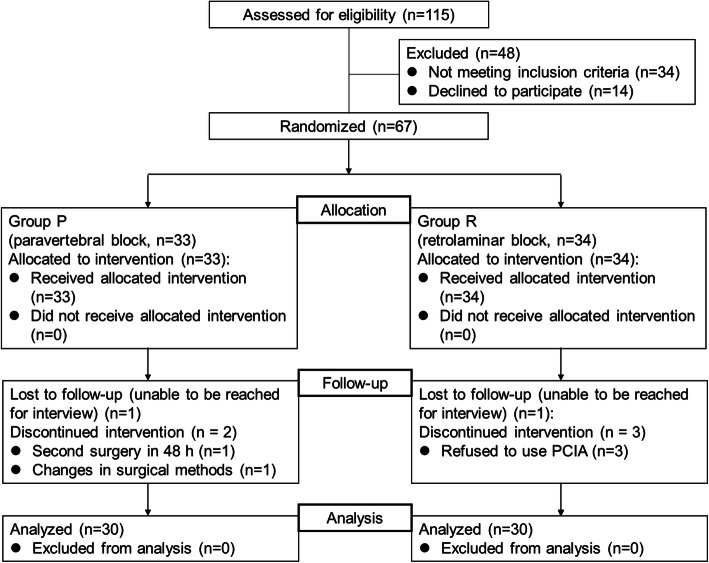
Patient inclusion and exclusion process. PCIA, patient-controlled intravenous analgesia

This prospective, randomized clinical study was approved by the institutional ethics committee of the National Cancer Center/National Clinical Research Center for Cancer/Cancer Hospital, Chinese Academy of Medical Sciences and Peking Union Medical College (2020080419244502), and registered in the Chinese Clinical Trial Registry (ChiCTR2100044060). Written informed consent was obtained from all patients. A total of 115 patients with lung cancer with an American Society of Anesthesiologists (ASA) physical status of I or II, aged 18 to 64 years old, who were selected for UVATS between March 2021 and July 2021 were recruited into this study (Fig. 1). All patients were able to communicate well and understood how to evaluate their pain score at rest or during coughing. The major exclusion criteria were as follows: infection at the site of injection for ultrasound-guided PVB or RLB; peripheral neuropathy; coagulation disorder; morbid obesity (body mass index (BMI) more than 40 kg/m^2^); allergy to ropivacaine; greater than first-degree heart block; hypertension; bradycardia (heart rate (HR) less than 60 beats per min); pregnancy; clinically significant cardiovascular, pulmonary, hepatic or renal disease; psychiatric illness that would interfere with the pain score assessment; and analgesic drug use within 1 week before surgery. During the preoperative interview, patients were taught how to evaluate their pain score using the numerical rating scale (NRS).

### Randomization and block procedures

**Fig. 2:**
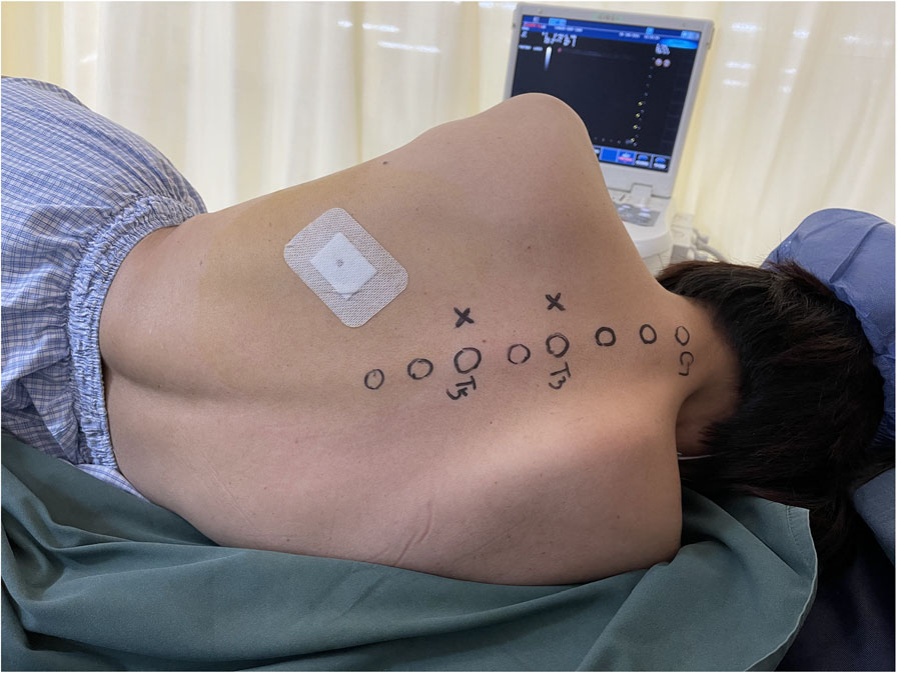
The T3 and T5 spinal segments where ultrasound-guided PVB or RLB was to be performed

Before the study, every patient was trained on how to use the patient-controlled intravenous analgesia (PCIA) pump and instructed on using the NRS, with a score of 0 indicating no pain and a score of 10 indicating the worst imaginable pain. Upon arrival in the nerve block room, the patient was placed in a lateral decubitus position with the operative side up, and standard ASA monitoring, including five-lead electrocardiography and monitoring of the HR, respiratory rate (RR) and pulse oxygen saturation (SpO_2_), was applied. All patients received oxygen through a mask. Sedation and analgesia were provided by intravenous administration of midazolam (1 to 2 mg) and sufentanil (5 to 10 μg), which were titrated to patient comfort during the whole nerve block procedure. For all patients, a high-frequency linear ultrasound transducer (4–13 MHz, Esaote MyLab 25 Gold, Genoa, Italy) was used to identify the order of the laminae of thoracic vertebrae and paravertebral spaces. After confirmation of satisfactory ultrasound visualization of both potential block sites, patients were randomized using a computer-generated list and opaque, sealed envelopes to one of two treatment groups: (1) ultrasound-guided PVB (group P) or (2) ultrasound-guided RLB (group R). Patients were blinded to the treatment group allocation (Fig. 2).

**Fig. 3:**
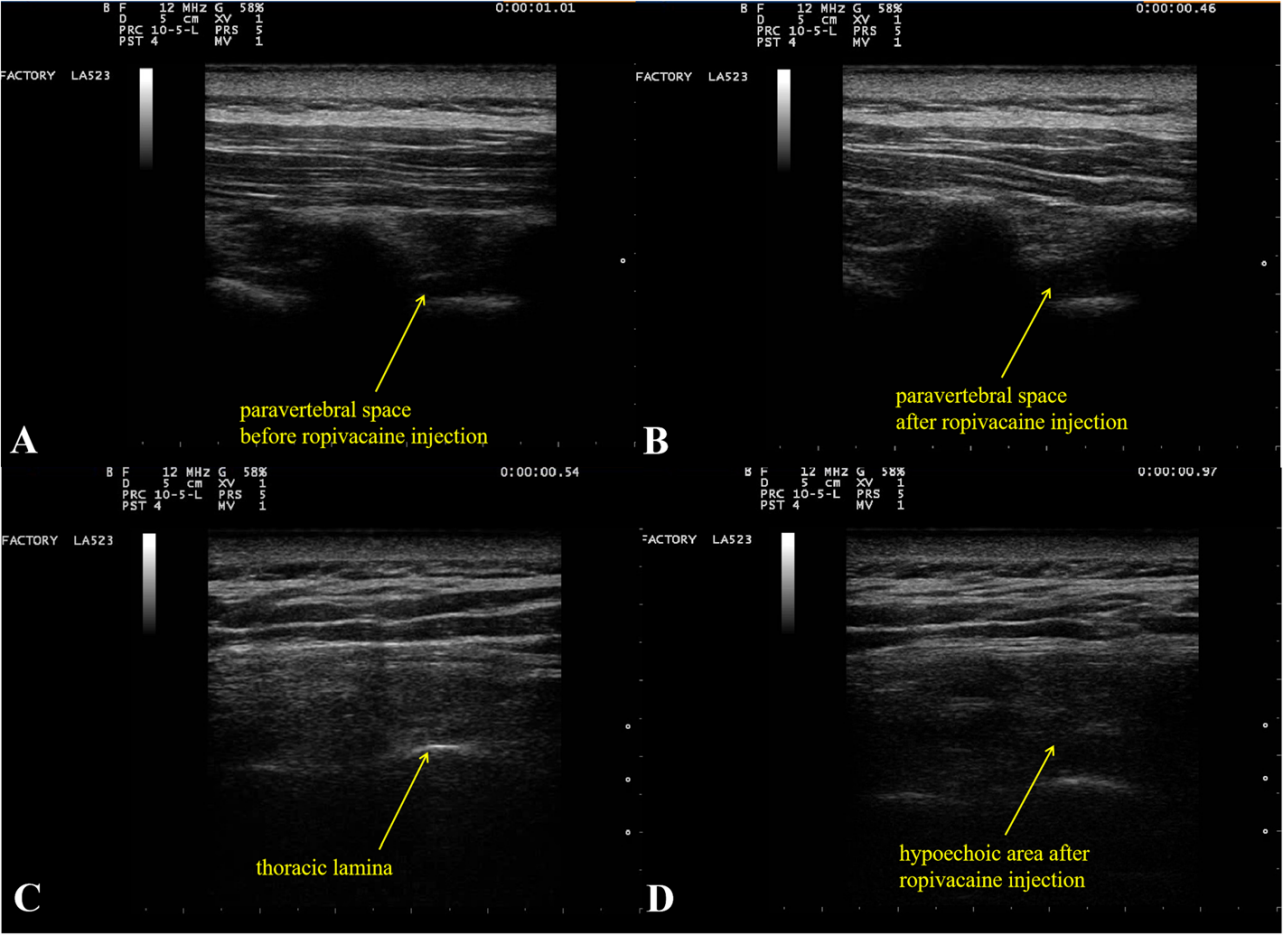
Ultrasound images of PVB and RLB. A shows an ultrasound image of the paravertebral space before the injection of ropivacaine. B shows that the paravertebral space widened after the injection of 15 ml of ropivacaine. C shows an ultrasound image of the thoracic lamina before the injection of ropivacaine. D shows the hypoechoic area formed by the injection of ropivacaine above the lamina

Under ultrasound guidance, after a skin wheal was raised using 2 ml of 1% lidocaine, an 80-mm, 22-gauge block needle (Stimuplex® D Plus; B Braun, Melsungen, Germany) was inserted in-plane in a caudad-to-cephalad direction. In addition, 0.5% ropivacaine was injected at the appropriate level(s) after negative aspiration (15 mL for each level on the operative side) (Fig. 3). For both PVB and RLB, a two-level injection technique was performed at T3 and T5. The nerve block was considered successful if, within 30 min, the patient experienced decreased sensation to pinprick at least from the ipsilateral third to sixth thoracic dermatomes at the level of the anterior axillary line.

### Intraoperative and postoperative management

General anesthesia was induced with 0.05 mg/kg midazolam, 1.5–2.5 mg/kg propofol, 0.2–0.4 μg/kg sufentanil and 0.6 mg/kg rocuronium. General anesthesia was maintained with desflurane, sufentanil and rocuronium. Intraoperative sufentanil was administered at the discretion of the blinded anesthesia team based on cardiovascular responsiveness to noxious stimuli in order to maintain systolic blood pressure within ±20% of baseline. When the patient’s systolic blood pressure increased by more than 20% from the baseline value, 0.1 μg/kg sufentanil was administered intravenously. After 10 min, if the systolic blood pressure was still higher than 120% of the baseline value, 0.1 μg/kg sufentanil was again administered intravenously. Ten minutes later, if the systolic blood pressure was still higher than 120% of baseline after two consecutive sufentanil boluses, 0.5 mg of nicardipine was administered until the systolic blood pressure decreased to baseline ±20%. During the operation, the velocity of fluid infusion was maintained at 6 mL/(kg.h).

**Fig. 4:**
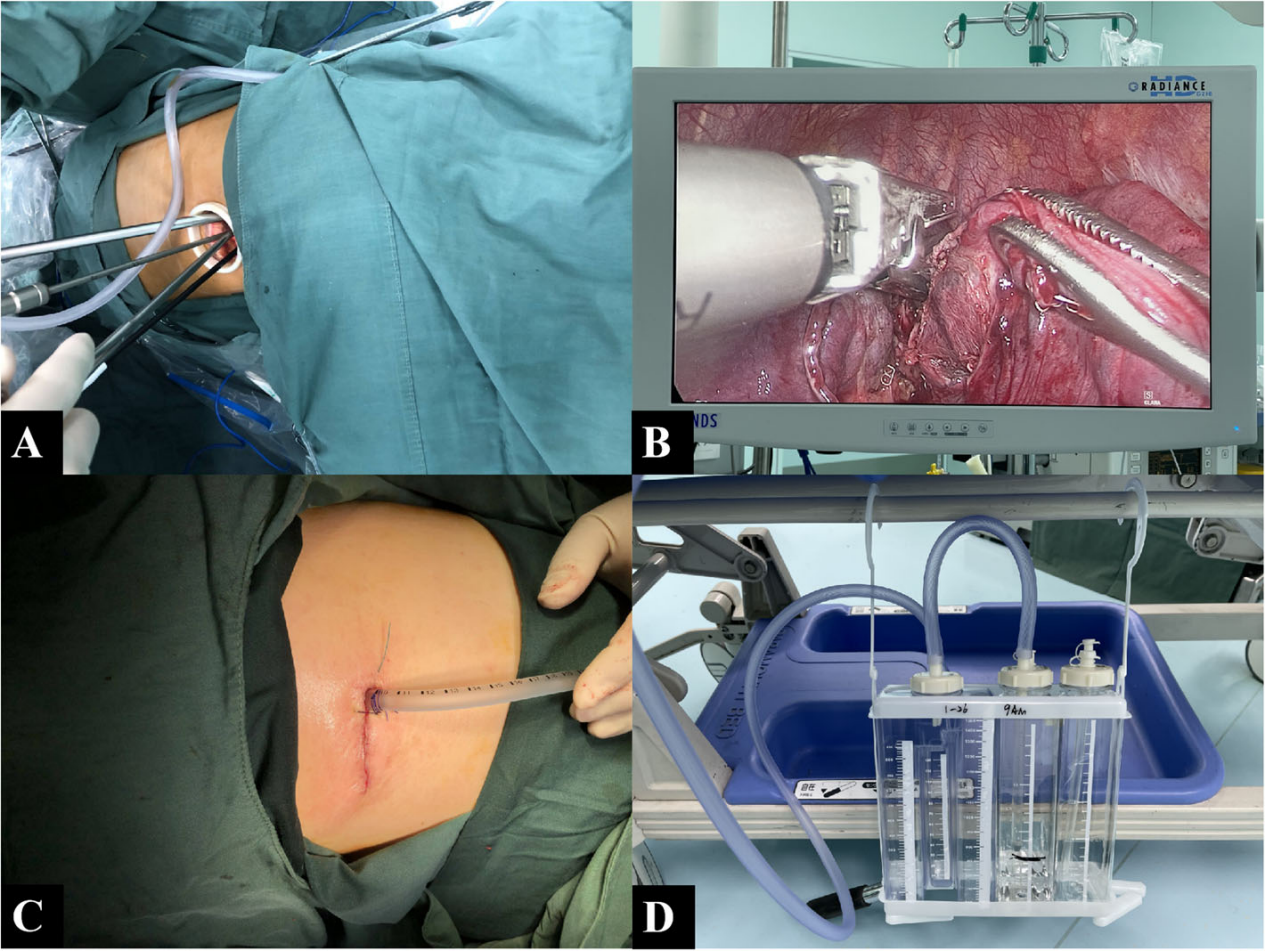
The 5-cm-long surgical incision was made in the fourth intercostal space at the anterior axillary line (A). B shows the surgical field of thoracoscopic lobectomy. A chest drain was placed at the edge of the incision at the end of the surgery (C). D shows the chest drainage system

After disinfecting the skin in the surgical area, a 5-cm-long incision in the fourth or fifth intercostal space at the anterior axillary line was made (Fig. 4). At the conclusion of the surgical procedure, a chest drain was placed at the edge of the incision (Fig. 4). At the end of the surgery, an intravenous analgesic pump was applied. The PCIA protocol was programmed with 2.5 μg/kg sufentanil diluted to 100 mL (bolus, 1.5 mL; lockout time interval, 10 min; 1 h limit, 9 mL without any baseline infusion). PCIA was administered when the NRS score was ≥4 or at the request of the patient. After surgery, patients were extubated, taken to the postanesthesia care unit (PACU) and received by a nurse anesthetist blinded to the randomization. In addition, a standard PACU care procedure was followed.

### Outcomes

The primary outcome was the NRS score within the 48-h period after surgery. The intraoperative sufentanil consumption and total postoperative sufentanil consumption within the 48-h period were also recorded, along with the time at which the patient first required analgesics after nerve block. Before the nerve block procedure (baseline) and at 3 h, 6 h, 12 h, 24 h, 36 h and 48 h after surgery, the patient’s mean arterial pressure (MAP), HR, and NRS scores at rest and during coughing were all assessed. Adverse events, such as bradycardia (HR less than 60 beats per min), hypotension (systolic blood pressure less than 90 mmHg or more than 20% lower than baseline), hypoxemia (SpO_2_ less than 90%), respiratory depression (RR less than 10 breaths per min lasting for more than 10 min), pruritus, neurotoxicity, backache, nausea and vomiting, were recorded after surgery. Bradycardia was treated with intravenous boluses of 0.5 mg of atropine. Hypotension was treated with 6 mg of ephedrine and 6 ml/kg normal saline; the same doses were repeated as required. Hypoxemia was treated with inhalation of oxygen through a face mask. Respiratory depression was treated with naloxone and oxygen until the respiratory rate was greater than 15 breaths per min.

In addition, adverse events related to block procedures were recorded. Pneumothorax and/or hydropneumothorax were indicated when patients presented with dyspnea. Physical exam revealed notably decreased breathing sounds and hyperresonance to percussion of the ipsilateral chest [16, 17]. In addition, video-assisted thoracoscopy confirmed that local anesthetic liquid had been injected into the chest cavity. After the nerve block procedure, new symptoms, such as weakness, pain, tingling, paresthesia or numbness in the skin of the corresponding innervated area, indicated nerve injury. Thoracic nerve root pain is often described as burning or sharp, stemming from the back and traveling to other parts of the body connected to the damaged nerve. If the patient had the symptoms described above, further MRI scans or CT scans was performed [18–20]. In this study, bleeding complications included the occurrence of vascular puncture, active bleeding, or hematoma formation caused by paravertebral block or retrolaminar block [21]. Bleeding complications were identified when blood was drawn back into the syringe during the nerve block operation or a chest wall hematoma was observed under thoracoscopy. The criteria for nerve block-related superficial soft tissue infection were swelling along the needle placement track, local tenderness along the needle placement track, fever (> 38.0 °C), and leukocytosis (> 12/nl or C-reactive protein (CRP) > 20 mg/l) [22]. The criteria for nerve block-related abscess or deep tissue infection were evidence of an abscess or fluid collection consistent with an infectious process by imaging or surgical exploration within 30 days after peripheral nerve block needle placement, fever (> 38.0 °C), positive culture from surgical exploration or puncture, leukocytosis (> 12/nl or CRP > 20 mg/l), local tenderness, focal back pain, and neurological deficit [22].

### Statistical analysis

The primary outcome was the pain score at rest within the 48-h period after surgery. In our preliminary study conducted in 10 adult patients (5 in each group), the mean NRS score at rest within the 48-h postoperative period was 2.0 ± 1.6 and 3.8 ± 1.9 in group P and group R, respectively. We hypothesized that ultrasound-guided PVB would reduce the NRS score compared with RLB. PASS version 11.0.7 (PASS, NCSS, LLC, USA) for Windows was used to calculate the sample size. Student’s t-test was selected, and the group allocation ratio was equal. The hypothesized means of the NRS scores were 2.0 and 3.8, and the standard deviations (SDs) were 1.6 and 1.9, respectively. Then, we calculated that a sample of 27 patients would provide 90% power at a two-sided alpha level of 0.05. Ultimately, we recruited 30 patients in each group for a total of 60 patients considering the possibility of dropout and loss to follow-up.

Continuous variables are presented as the mean ± SD or median (25th to 75th percentiles), and categorical data are presented as numbers and percentages. Normality was tested by Kolmogorov-Smirnov analysis. Student’s t-test or Mann-Whitney U test was used for analysis of the NRS score, intraoperative sufentanil consumption, total postoperative sufentanil consumption and duration of analgesia. For analysis of the MAP and HR data, repeated-measures ANOVA with Bonferroni correction was used. To analyze rescue flurbiprofen axetil, the incidence of adverse effects, Fisher’s exact test was used. All data were processed by IBM SPSS Statistics 21.0 (IBM, Inc., New York, NY). A 2-sided *p* value less than 0.05 was considered statistically significant.

## Results

A total of 115 patients were recruited to participate in this study from March 2021 to July 2021. In all, 48 patients were ineligible because they did not meet the inclusion criteria or declined to participate, and 7 patients were excluded from the trial because of nonadherence to the study protocol (2 patients were lost to follow-up; 1 patient underwent secondary emergency surgery in 48 h; 1 patient temporally underwent a two-port operation instead of a uniportal operation during surgery; and 3 patients refused to use PCIA after surgery). Ultimately, 60 patients were randomized and completed the study protocol (group P: *n* = 30; group R: n = 30). The Consolidated Standards of Reporting Trials flow diagram depicts participant progression through the study phases (Fig. 1).

Ultimately, a total of 60 patients were included in the analysis in this study. There were no significant differences in the patient characteristics between the groups (Table 1). Additionally, there were no differences in the surgical method, duration of surgery or duration of anesthesia between the two groups (Table 1).

**Table 1:** Demographic data and surgical and anesthetic characteristics

	Group P	Group R	***P*** value
Age (years)	53.7 ± 14.0	55.3 ± 11.8	0.630
BMI (kg/m^2^)	24.3 ± 3.0	23.2 ± 3.5	0.602
Sex (F/M)	22/8	25/5	0.532
ASA classification (I/II)	4/26	6/24	0.488
Surgical method (uniportal video-assisted lobectomy/uniportal video-assisted wedge resection)	20/10	17/13	0.434
Duration of surgery (min)	112.8 ± 35.5	116.23 ± 49.9	0.762
Duration of anesthesia (min)	138.5 ± 39.3	142.6 ± 49.6	0.724
Intraoperative sufentanil consumption (μg)	22.60 ± 2.83	26.64 ± 3.54	< 0.001
Total postoperative sufentanil consumption (μg)	21.52 ± 8.48	38.91 ± 13.27	< 0.001
Rescue flurbiprofen axetil (used/not used)	2/28	4/26	0.671
Rescue meperidine (used/not used)	0/30	0/30	–

Data are expressed as the mean ± SD for numbers. There were no significant differences between the groups. *BMI* body mass index, *ASA* American Society of Anesthesiologists, *F* female, *M* male, *P* paravertebral block, *R* retrolaminar block.

**Fig. 5:**
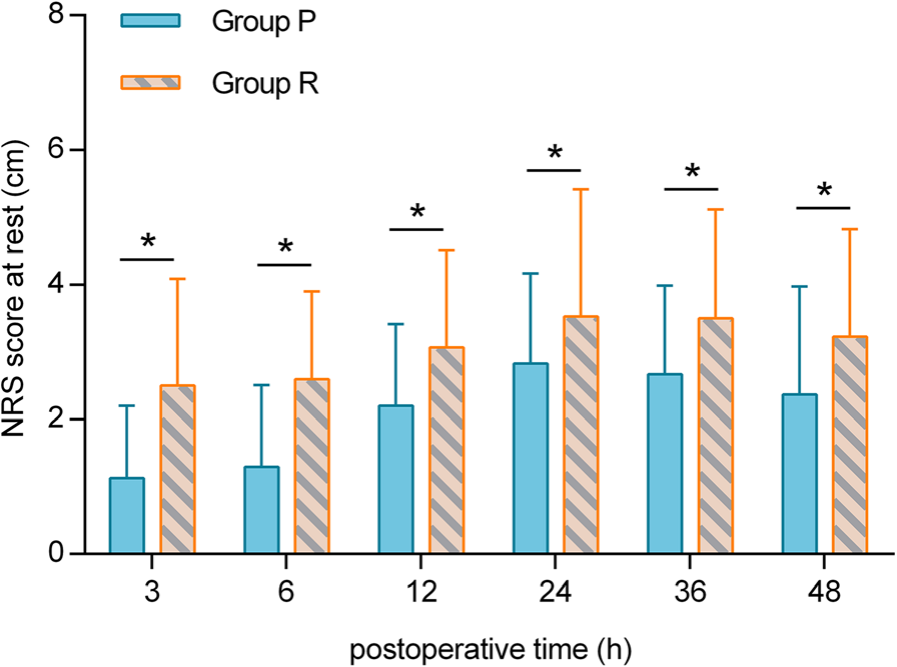
Postoperative pain severity NRS score at rest (in cm) at 3, 6, 12, 24, 36 and 48 h postoperatively. P, paravertebral block; R, retrolaminar block; NRS, numerical rating scale. ^*^P < 0.05

**Fig. 6:**
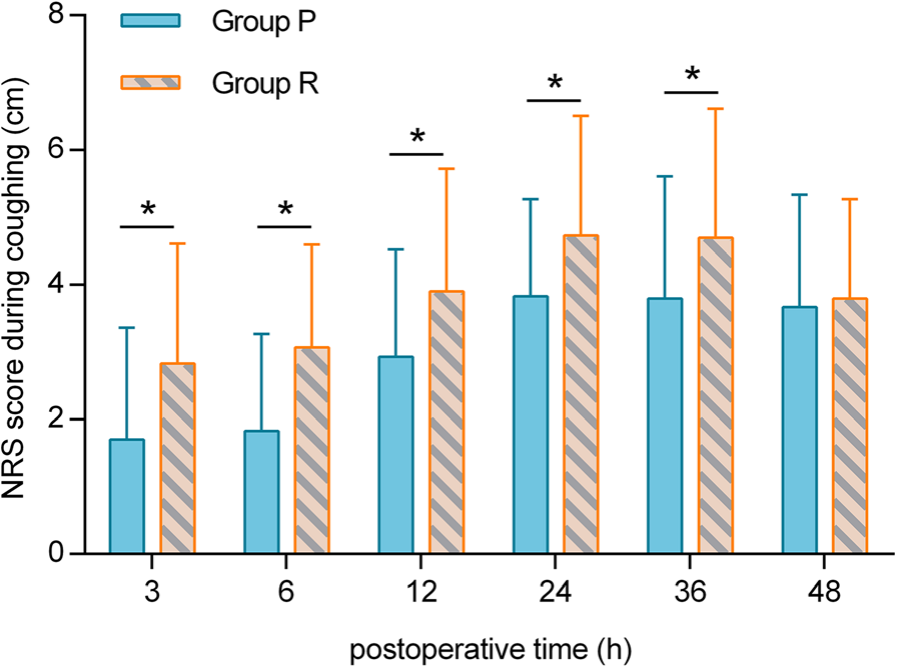
Postoperative pain severity NRS score during coughing (in cm) at 3, 6, 12, 24, 36 and 48 h postoperatively. P, paravertebral block; R, retrolaminar block; NRS, numerical rating scale. ^*^P < 0.05

At 3, 6, 12, 24, 36 and 48 h postoperatively, the NRS score at rest in group R was higher than that in group P (*p <* 0.05) (Fig. 5). At 3, 6, 12, 24 and 36 h postoperatively, the NRS score during coughing in group R was higher than that in group P (*p* < 0.05) (Fig. 6). The intraoperative sufentanil consumption and total postoperative sufentanil consumption in group P were significantly lower than those in group R (*p* < 0.001) (Table 1).

**Fig. 7:**
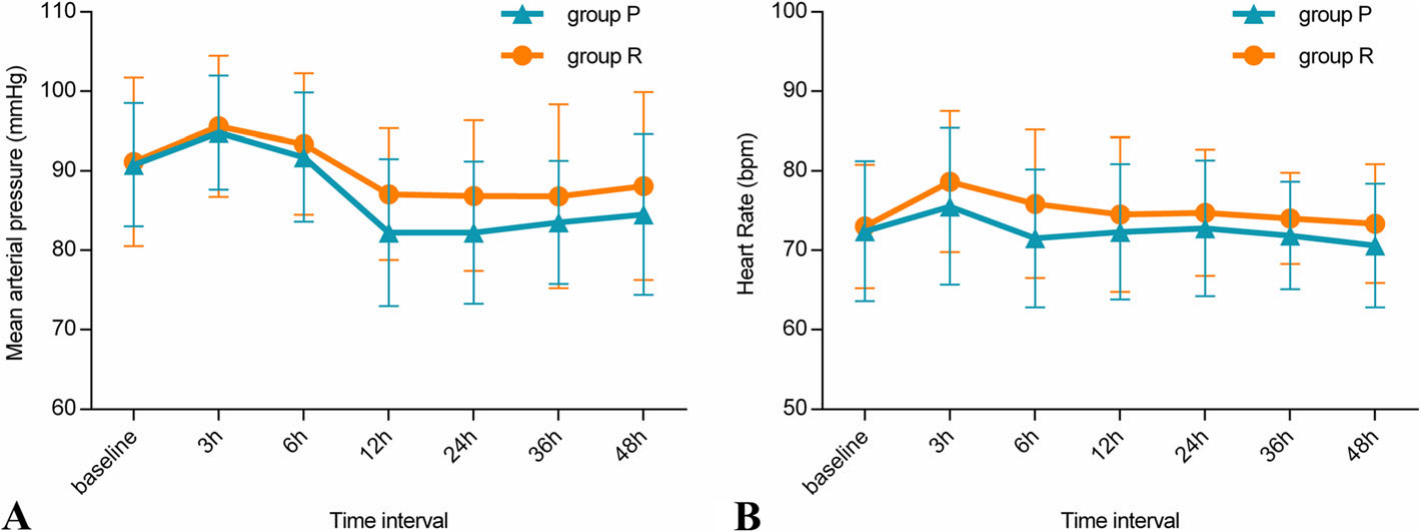
A shows the mean arterial pressure (MAP) changes at different times in each group. In both groups, the MAP showed significant changes over time, with ^*^P < 0.001. B shows the heart rate (HR) changes at different times in each group. In both groups, the HR showed significant changes over time, with ^*^P < 0.05. P, paravertebral block; R, retrolaminar block

The MAP was significantly different at different time points in each group (*p <* 0.001). Similarly, the discrepancy in the HR at different time points was statistically significant (*p* < 0.01). However, no significant difference in the MAP or HR was observed between group P and group R (*p* = 0.251 and *p* = 0.079, respectively) (Fig. 7).

Table 2 shows the number of patients experiencing adverse events. There was no significant difference in the incidence of bradycardia, hypotension, hypoxemia, respiratory depression, vomiting, pruritus, dizziness or neurotoxicity between group P and group R. However, the incidence of nausea in group R was higher than that in group P (*p* < 0.05).

**Table 2:** Incidence of adverse events

Adverse events	Group P	Group R	***P*** value
**Adverse events related to block procedures**			
Pneumothorax or hydropneumothorax	1 (3)	0 (0)	1.000
Nerve injury	0 (0)	0 (0)	–
Bleeding complications	0 (0)	0 (0)	–
Infection	0 (0)	0 (0)	–
**Other adverse events**			
Bradycardia	0 (0)	0 (0)	–
Hypotension	0 (0)	0 (0)	–
Hypoxemia	0 (0)	0 (0)	–
Respiratory depression	0 (0)	0 (0)	–
Nausea	1 (3)	8 (27)	0.026
Vomiting	1 (3)	4 (13)	0.353
Pruritus	0 (0)	0 (0)	–
Dizziness	2 (7)	4 (13)	0.671
Neurotoxicity	0 (0)	0 (0)	–

The incidence of adverse effects data are expressed as numbers and percentages. *P* paravertebral block, *R* retrolaminar block.

## Discussion

In this study, we compared the analgesic effect of ultrasound-guided PVB and RLB after UVATS, and the NRS score within the 48-h period after surgery was the primary outcome. We found that compared with RLB, PVB could provide better analgesia both at rest and during coughing after surgery. Additionally, postoperative opioid consumption was deceased by PVB compared to RLB, and the incidence of nausea was lower after PVB than after RLB.

To date, only one study has reported the use of PVB and RLB for postoperative analgesia after thoracic surgery [23]. However, there were significant differences between the previous study and this study in terms of research design. First, in a previous study conducted by Sugiyama et al., 0.5% ropivacaine was used for PVB, while 0.25% ropivacaine was used for RLB. Moreover, the volume of ropivacaine used for the two nerve block techniques was also quite different. Therefore, both the concentration and volume of local anesthetics used in the two nerve block techniques were completely different. A number of studies have confirmed that when the same local anesthetic is used for the same nerve block technique, different concentrations or volumes produce different analgesic effects [24–26]. Therefore, in the previous study conducted by Sugiyama et al., although the conclusion was that the analgesic effect of PVB was better than that of RLB after thoracic surgery, it was likely that the difference in the concentration and volume of local anesthetic drugs contributed to the different analgesic effects of the two nerve block techniques. In the previous study conducted by Sugiyama et al., it was difficult to distinguish whether the different analgesic effects were due to the different characteristics of the two nerve block techniques themselves or to the different concentrations or volumes of local anesthetic agents used. In contrast, in this study, the same concentration and volume of ropivacaine was used in either PVB or RLB to compare the analgesic effects of the two nerve block techniques. Therefore, in this study, the analgesic effects of these two nerve block techniques were comparable, and the conclusion was more convincing.

In addition, previous studies have indirectly compared the analgesic effects of PVB and RLB. After UVATS, Wang et al. confirmed that ultrasound-guided PVB could effectively alleviate postoperative pain and reduce postoperative opioid consumption [6]. Nagane et al. found that ultrasound-guided RLB effectively relieved postoperative pain after pulmonary lobectomy [15]. Furthermore, previous researchers have compared the analgesic effects of RLB and another similar thoracic nerve block, i.e., erector spinae plane block (ESPB). In addition, the analgesic effects of PVB and ESPB have been compared in thoracoscopic surgery. Sotome et al. found that RLB was equivalent to ESPB for analgesia after breast surgery using 20 ml of 0.375% levobupivacaine [27]. Comparatively, Turhan et al. found that ultrasound-guided PVB could produce better analgesia and reduce opioid consumption than ESPB after VATS [28]. In addition, Chen et al. confirmed that compared with ultrasound-guided ESPB, ultrasound-guided PVB provided superior analgesia after thoracoscopic surgery [29]. Consistent with the aforementioned evidence, the results of this study suggest that ultrasound-guided PVB provided superior analgesia within 48 h after UVATS than ultrasound-guided RLB.

In this study, the target injection site of ultrasound-guided RLB was the posterior surface of the thoracic vertebral lamina [30]. Compared with PVB, the exact mechanism of analgesia of RLB was not completely clear. As revealed by Yang et al. in a cadaveric study, the probable mechanism of action for RLB was the spread of local anesthetics into the paravertebral space via the superior costotransverse ligament [14]. The anatomical basis was that although the superior costotransverse ligament was well developed at the T7 to T10 levels, it was rudimentary at the T1 to T6 levels [31]. In this study, RLB was performed at the T3 and T5 levels. Therefore, the ropivacaine solution was able to infiltrate easily into the paravertebral space. Ultimately, the anterior and posterior branches of the thoracic spinal nerves were blocked mainly by the indirect effect of RLB, and an analgesic effect was induced [32]. In contrast, PVB produces analgesia by directly depositing local anesthetics into the paravertebral space to block the ventral ramus and dorsal ramus of the spinal nerve root [33]. Compared with the indirect mechanism of RLB, the direct mechanism of PVB probably results in superior analgesia.

In this study, ultrasound-guided PVB exhibited an opioid-sparing effect compared to ultrasound-guided RLB. This can probably be attributed to the superior analgesic effect of PVB. In a previous clinical study, Turhan et al. noted that ultrasound-guided PVB reduced morphine consumption after thoracoscopic surgery [28]. Similarly, in thoracoscopic radical lung cancer surgery, ultrasound-guided PVB reduced postoperative oxycodone consumption [34]. In addition, Chen et al. confirmed that ultrasound-guided PVB significantly reduced cumulative morphine consumption after thoracoscopic surgery [29]. The findings of the aforementioned previous studies are consistent with the results of this study. In addition, in this study, the incidence of nausea was lower with ultrasound-guided PVB than with RLB, which can probably be attributed to the reduced postoperative sufentanil consumption. As noted by Smith et al., opioids can induce nausea with or without vomiting, and this distressing symptom is related largely to the dose of opioid administered [35]. Similarly, Rivedal et al. found that PVB was associated with decreased opioid consumption and a decreased incidence of nausea and vomiting after breast surgery [36].

Compared with PVB, RLB is theoretically safer due to the anatomical avoidance of the pleura and vascular structures [37]. Additionally, because of the superficial anatomical location, RLB is technically easier to perform for clinical practitioners, especially for inexperienced novices [38]. In this study, only one patient suffered pneumothorax or hydropneumothorax during the ultrasound-guided PVB procedure. The reason for the low incidence of pneumothorax in this study might be that the anesthesiologists performing the PVB procedure were all attending doctors with a great deal of prior experience with the PVB technique. However, for anesthesiologists with less experience performing PVB, the risk-to-benefit ratio might be better with RLB, which is more superficial than PVB.

There are several limitations to this study. First, this was a single-center, small-sample, exploratory clinical study, and further multicenter, large-sample clinical trials are needed to further confirm the conclusion of this study. Second, the type, concentration and volume of local anesthetic used in this study were specific. The results might not be suitable for generalization to include the use of different local anesthetics or volumes. Third, the surgical method observed in this study was UVATS. If the surgical procedure is changed to three-port thoracoscopic surgery or open thoracotomy, an inconsistent conclusion might be drawn. Therefore, further investigations in different surgical approaches for lung cancer are needed to verify the conclusions of this study.

## Conclusions

In conclusion, the results of this study suggest that in patients with lung cancer undergoing UVATS, ultrasound-guided PVB with 0.5% ropivacaine provides better analgesia and results in less nausea than ultrasound-guided RLB. Compared with ultrasound-guided RLB, ultrasound-guided PVB seems to be a better technique for analgesia in UVATS.

## Data Availability

The datasets generated or analyzed during the current study are available from the corresponding author on reasonable request.

## Abbreviations

UVATS: Uniportal video-assisted thoracoscopic surgery
PVB: Paravertebral block
RLB: Retrolaminar block
ASA: American Society of Anesthesiologists
BMI: Body mass index
HR: Heart rate
NRS: Numerical rating scale
PCIA: Patient-controlled intravenous analgesia
RR: Respiratory rate
SpO_2_: Pulse oxygen saturation
PACU: Postanesthesia care unit
MAP: Mean arterial pressure
